# Chlamydia trachomatis restricts signaling through NOD2 until late in the pathogen’s developmental cycle

**DOI:** 10.1101/2025.05.28.656691

**Published:** 2025-05-30

**Authors:** Grace Overman, Iris Loeckener, Zachary Williford, Sung Davis, Aissata Diallo, Beate Henrichfreise, George W. Liechti

**Affiliations:** 1 Department of Microbiology and Immunology, Uniformed Services University, Bethesda, MD, United States of America.; 2 Henry Jackson Foundation for the Advancement of Military Medicine, Bethesda, MD, United States of America.; 3 Institute for Pharmaceutical Microbiology, University of Bonn, Bonn, Germany.

**Keywords:** *Chlamydia*, Innate Immunity, peptidoglycan, NOD2, pathoadaptation, persistence

## Abstract

Pathogenic chlamydial species restrict their peptidoglycan (PG) to the division septum of their replicative forms. PG is a microbe-associated molecular pattern (MAMP) and two of its major pattern recognition receptors in human cells are nucleotide-binding oligomerization domain-containing proteins 1 and 2 (NOD1 and NOD2, respectively). It has been proposed that this unique morphological feature is evidence of pathoadaptation by the microbe, permitting PG-dependent cell division while also reducing the bacterium’s recognition by innate immune receptors. *Chlamydia trachomatis*-infected cells activate NOD1 signaling within 8–12 hours of exposure to the bacterium, roughly coinciding with the microbe’s transition from its infectious to replicative forms. Here we report that, unlike NOD1 signaling, *Chlamydia*-induced NOD2 signaling does not occur until later in the pathogen’s developmental cycle. Both *C. trachomatis* and the related murine pathogen *Chlamydia muridarum* signal late in infection in HEK293 reporter cell lines expressing either human or murinederived NOD2 receptors. NOD2 signaling can be modulated by disruption of the chlamydial amidase enzyme, AmiA_CT_, interrupting the microbe’s developmental cycle, and inducing RB lysis with inhibitors of lipooligosaccharide or peptidoglycan biosynthesis / assembly. These results mirror prior observations with Chlamydia-induced TLR9 signaling, leading us to hypothesize that Chlamydia-induced NOD2 signaling results from RB lytic events that occur sporadically during the RB to EB transition. Given our finding that pre-treating cells with NOD2-stimulatory ligands reduces chlamydial inclusion size, we hypothesize that the microbe preferentially degrades its PG during development to reduce the generation of NOD2 ligands, at the cost of enhancing NOD1 signaling.

## INTRODUCTION

*Chlamydia trachomatis* is the leading cause of bacterial sexually transmitted disease and infectious blindness in the world, with over 100 million estimated cases of sexually transmitted infection^1^ and ~100 million people still at risk of trachoma-induced blindness^2^. This obligate, intracellular microbe has evolved a number of pathoadaptive means of circumventing the human immune system at the cellular level, and as a result, clearance of the organism is uncommon without intervention. Though this pathogen is susceptible to many commercial antibiotics, the estimated burden within the global population remains high due to the large majority of infections being asymptomatic^3,4^, enabling the organism to spread in the absence of effective screening and treatment.

One of the adaptations made by chlamydial species to life inside of host organisms is the utilization of a biphasic developmental cycle^5^. Infectious forms, termed Elementary bodies (EBs) have reduced metabolic rates^6^ and utilize lipooligosaccharide and gDNA with significantly reduced immunostimulatory properties^7–11^. Upon entering into host cells, chlamydial EBs transition into Reticulate bodies (RBs), and begin replicating via a unique cell division process^12^ that is dependent on the synthesis and turnover of peptidoglycan (PG)^13–15^. In most other bacterial species, to include some chlamydial-related organisms^16^, PG is a major component of the bacterial cell wall (termed a sacculus) that confers strength and rigidity to bacterial cells, while also allowing microbes to maintain or alter their overall shape^17–19^. Pathogenic chlamydial species lack sacculi, but synthesize PG at their division planes^13,15,20^. Paradoxically, because they lack FtsZ^21^, the major organizer of septal PG in almost all bacterial species^22^, they utilize the PG synthesis machinery primarily associated with sacculus construction in other bacterial species^15,23–29^. While encoding two PG synthase complexes primarily associated with either side-wall^30^ or septal^31^ PG biosynthesis, *C. trachomatis* utilizes both to modulate the dimensions of its ‘PG ring’ during the initial and terminal stages of its division process^32,33^.

It has been proposed that the absence of PG sacculi in chlamydial species is evidence of pathoadaptation by these organisms^13,15,34,35^, due to PG being a major immunostimulatory molecule that is recognized by a wide range of host cellular receptors^36^. As PG is only present in the organism’s replicative forms and in relatively low abundance when compared to the amount required to generate sacculi, it stands to reason that reducing the abundance of PG-derived muropeptides would directly impact *Chlamydia*-host cell interaction(s). This hypothesis is supported in part by the characterization of a rudimentary PG-recycling pathway encoded by *C. trachomatis* that enables the reabsorption of meso-diaminopimelic acid (mDAP)– containing muropeptides^37,38^, further reducing its immunostimulatory profile.

The two most well-studied immunoreceptors for bacterial PG recognition are the NOD-like receptors (NLRs) NOD1 and NOD2^39^. NOD1 recognizes PG-derived peptides containing mDAP^40^ while NOD2 primarily recognizes peptides containing N-acetylmuramic acid (MurNAc), the principle component of NOD2 / CARD15 stimulatory ligands^41^. While it has been over a decade since PG was first demonstrated to be present in these organisms^13^, the degree to which these receptors play a role during Chlamydia infections is still in question^42^. Correlative studies examining patient cohorts have noted an association between NOD1 functionality and bacterial clearance / disease pathology^43,44^ while a less robust association has been observed between reduced NOD2 functionality and an increased risk of tubal pathology subsequent to chlamydial infection^45^. Both receptors are expressed throughout the female reproductive tract^46,47^, with some evidence that expression of NOD2 (but not NOD1) is impacted by phases of the menstrual cycle^48^. NOD1 induction has been demonstrated to have a significant role in inducing inflammation in vitro^49–51^, and murine studies have linked both receptors to Endoplasmic Reticulum Stress-Induced Inflammation during chlamydial infection and NOD1/2-deficient mice exhibit a reduction in clearance rates^52,53^, though it is presently unclear if these phenotypes are associated with ligand-dependent or ligand-independent NOD1/2 signaling^54^.

We have previously reported that PG can be visualized in cells infected with *C. trachomatis* as early as 8 hours post-infection (hpi)^13^, coinciding with the transition of EBs to RBs and transcriptome data demonstrating the expression of chlamydial genes associated with PG biosynthesis and turnover^55^. NOD1 signaling can be observed within 24 hpi in cells infected with *C. trachomatis*, and we have previously demonstrated that treatment with various antibiotics, metal chelators, and host cell cytokines can impact the intensity of chlamydia-induced NOD1 signaling^56^. Interestingly, while NOD2-stimulatory ligands have been reported to be present in lysates generated from Chlamydia-infected cells^57^, no signaling was observed 24 hpi when reporter cells were infected directly, as noted by Packiam (personal communication, April 2014). Given our recent work demonstrating that TLR9 signaling is delayed in Chlamydia-infected cells and appears to be dependent on either bacterial lysis or transitioning between chlamydial forms^9^, we reasoned that this could potentially explain the discrepancy under these two conditions. We set out to establish the dynamics of Chlamydia-induced NOD2 signaling, and determine what factor(s) impact its amplitude and temporal kinetics.

## RESULTS

### *Chlamydia trachomatis* signals via NOD2 late in the pathogen’s developmental cycle.

**Figure 1.**
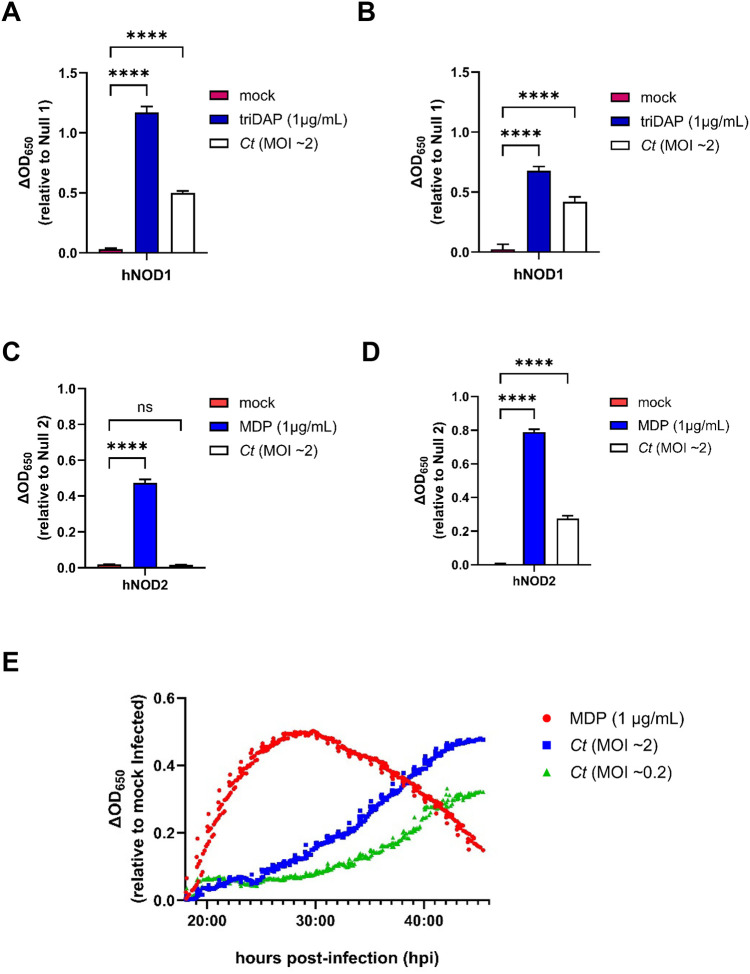
*Chlamydia trachomatis* signals via NOD2 late in the pathogen’s developmental cycle. SEAP activity was measured from the supernatants of hNOD1 and hNOD2 and Null1/2 HEK 293 reporter cells infected with *C. trachomatis* serovar L2 (strain Bu/434) at 24 **(A, C)** and 44 **(B, D)** hpi. Columns represent the mean value calculated for data acquired from three separate experiments (biological replicates) and error bars represent standard error of the mean. Groups were compared via one-way ANOVA with multiple comparisons. ****; p ≤ 0.0001, *; p ≤ 0.05, ns; not significant. **(E)** SEAP activity was measured in the supernatants of infected NOD2-expressing HEK 293 reporter cells grown in HEK-Blue^™^ Detection media. OD_650_ values for each well were assessed every ten minutes beginning at 18 hpi, and values plotted are relative to mock infected control wells on the same plate. Data is representative of three wells per condition on the same plate (technical replicates). Positive control (muramyl dipeptide; MDP) was added to control wells at 18 hpi.

We utilized a secreted embryonic alkaline phosphatase (SEAP) reporter system to compare the relative signaling of human (h)NOD1 and hNOD2 in cells infected with *C. trachomatis*. SEAP was detected in the supernatants of infected cells at elevated levels at 24 and 48 hours post infection (hpi) for NOD1 (Fig. 1A, B), however, NOD2 signaling appeared to be delayed as SEAP was only present at appreciable levels in cell supernatants at 48 hpi (Fig. 1C, D). In order to more closely follow the kinetics of *C. trachomatis*-induced NOD2 signaling, we conducted real-time monitoring of SEAP levels present in cell supernatants, beginning at 18hpi. Infected cells were incubated in HEK-Blue Detection cell culture medium, allowing hydrolysis of substrate by SEAP to be measured via microplate reader every 10 minutes for 28 hours. We found that SEAP begins to accumulate at detectable levels ~19–20 hpi and that activity began to accelerate in a MOI-dependent manner at ~24 hpi (MOI ~2) and ~30 hpi (MOI ~0.2) (Fig. 1E). As these observations roughly overlap with the pathogen’s transition from its replicative to infectious form, we concluded that NOD2 ligand release may be the result of *C. trachomatis* undergoing developmental phase conversion.

### Chlamydia-specific NOD2 signaling in HEK293 cells occurs as a result of the RB-to-EB conversion.

**Figure 2.**
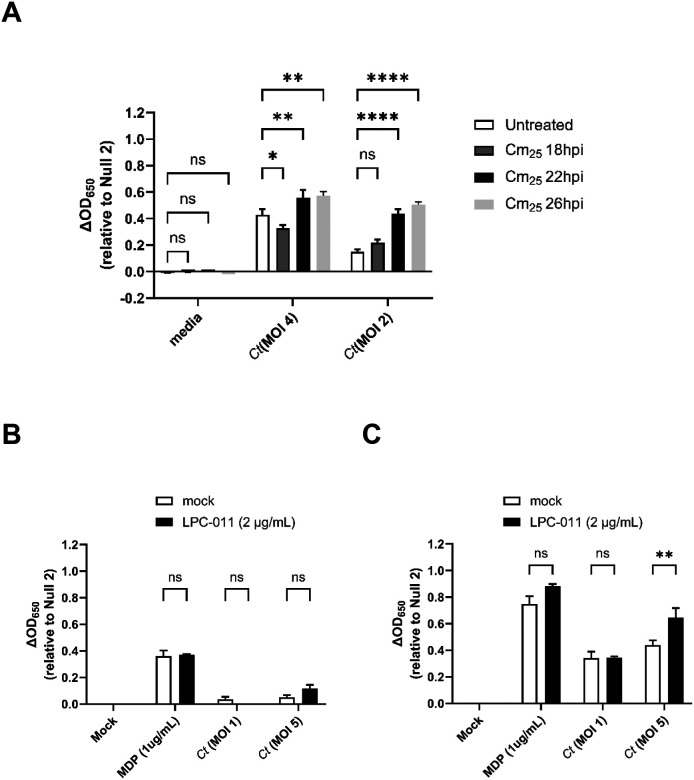
Chlamydia-specific NOD2 signaling in HEK293 cells occurs as a result of the RB-to-EB conversion. **(A)** *C. trachomatis*-infected hNOD2-HEK293 reporter cells were treated with chloramphenicol (25 ug/mL; Cm_25_) at the time points indicated and SEAP activity was measured at 44hpi. **(B,C)** The effects of the LOS inhibitor LPC-011 on *C. trachomatis*-induced NOD2 signaling at 24 **(B)** and 44 **(C)** hpi. All columns represent mean values calculated for data acquired from three biological replicates and error bars represent standard error of the mean. Groups were compared via two-way ANOVA with multiple comparisons. ****; p ≤ 0.0001, **; p≤ 0.01, *; p ≤ 0.05, ns, not significant.

We have previously reported that recognition of chlamydial species by TLR9 also occurs late in infection, and we proposed that chlamydial DNA is likely released due to random lytic events that occur during the transition from RBs to EBs^9^. To test whether interrupting the developmental cycle could prevent / dampen NOD2-stimulatory muropeptide release, we infected hNOD2 cells with *C. trachomatis*, and added chloramphenicol at various time points post infection to interrupt protein synthesis and subsequently prevent developmental form conversion. When protein synthesis was inhibited at 18 hpi, a time point prior to the RB-to-EB conversion^58^, NOD2 signaling decreased, and slowly increased when chloramphenicol was added at later and later time points (Fig. 2A). By contrast, when chlamydial lysis was enhanced during the RB-to-EB transition with the use of a LpxC inhibitor (LPC-011^59,60^), signaling was significantly increased, (Fig. 2B,C). This enhancement did not appear to significantly impact the timing of NOD2-ligand release, as signaling was only observed at the 44hpi time point and only when higher MOIs were used. These observations, in addition to the kinetics of Chlamydia-specific NOD2 signaling (Fig 1E), support the premise that NOD2 ligand release from *C. trachomatis* is likely a result of developmental form conversion.

### Knock-down of *amiA*_*Ct*_ impacts NOD2 signaling.

Assuming that RB lysis is the cause for the release of NOD2-stimulatory ligands in Chlamydia-infected cells, it is not readily apparent why the release of Chlamydia-specific, NOD1-stimulatory ligands is not similarly impacted. As mentioned previously, *C. trachomatis* encodes a rudimentary peptidoglycan recycling pathway that enables it to limit the amount of meso-DAP-containing peptidoglycan fragment release into the intracellular environment^37,38^, however, to date no pathway for the recycling of anhydroMurNAc has been characterized in any Chlamydia species.

We questioned whether the molecular process(es) of PG degradation employed by the pathogen potentially influence its NLR stimulatory potential. Chlamydia and chlamydia-related organisms make use of two separate degradation enzymes in order to remodel their septal PG during the initiation and termination of their division process: an amidase (AmiA) that cleaves the stem peptide from MurNAc^61^ and a lytic transglycosylase (SpoIID) that cleaves denuded glycan strands^62^. MurNAc alone is a poor stimulator of NOD2 receptors, and most stimulatory ligands require at least two amino acids of the PG stem peptide to be present, in the correct spatial isomerism, in order for MurNAc to bind sufficiently well to result in a signaling cascade^63^. We reasoned that under normal conditions inherent to the PG turnover found in *Chlamydia* species, sufficient amidase activity would effectively eliminate any and all amino acids attached to MurNAc, thus fundamentally skewing NLR signaling towards NOD1 (which is mDAP-specific^40^) and away from NOD2.

**Figure 3.**
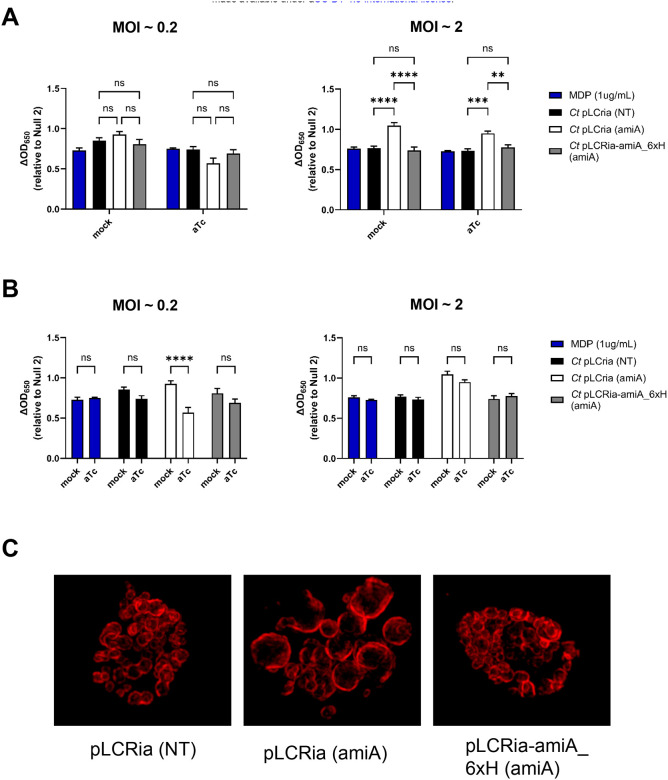
Knockdown of *amiA*_ct_ marginally impacts NOD2 signaling at lower MOIs. hNOD2-expressing HEK293 SEAP reporter cells were infected with *C. trachomatis* strains at MOIs of 2 and 0.2 containing either pLCria (NT), pLCria (*aimA*), or pLCria-amiA_6xHIS (*amiA*). Infections were carried out in the presence / absence of 1nM aTc and supernatants were tested for the presence for SEAP activity 48 hpi. All columns represent mean values calculated for data acquired from three biological replicates and error bars represent standard error of the mean. Groups were compared via two-way ANOVA with multiple comparisons. ****; p ≤ 0.0001, ns, not significant. Data are presented as comparisons between strains **(A)** and between conditions **(B). (C)** Representative images of *C. trachomatis* strains transformed with pLCRia, pLCRia (amiA), and pLCRia-amiA_6xH (amiA) plasmids under uninduced conditions 24hpi. Images are representative of 20 fields of view observed over two separate experiments.

In order to assess the impact of the chlamydial amidase on NOD2 signaling, we utilized a previously characterized CRIPRi-knockdown approach^64^ in order to gauge how reducing amidase activity would impact NOD signaling in chlamydia-infected cells. *C. trachomatis* strain -pL2 was transformed with either an empty plasmid; pLCria (NT), a knockdown construct; pLCria (*aimA*), or a knockdown construct with a HIS-tagged complementation allele; pLCria-amiA_6xHIS (amiA) (Dannenberg *et al*., in prep). Each strain was then tittered and used to infect hNOD2-expressing HEK293 reporter cells under either native or inducing conditions. Counter to our initial hypothesis, the transformant containing the AmiA knockdown plasmid appeared to exhibit slightly enhanced baseline NOD2 signaling when compared to control strains, a trend that reached statistical significance when higher MOIs were used (Fig. 3A, **right column**). When the three strains were compared using a lower MOI, the AmiA knockdown strain exhibited a decrease in NOD2 signaling (Fig. 3B, **left column**), a result that did not reach significance in either empty vector or complementation control strains. When examining the bacteria under non-induced conditions, RBs from the pLCria (*aimA*)-transformed strain appeared to be slightly enlarged (Fig. 3C), indicating that the presence of the knockdown plasmid itself was likely adversely impacting the size (and potentially the development) of the microbe.

### Inducers of persistence enhance Chlamydia-specific NOD2 signaling.

**Figure 4.**
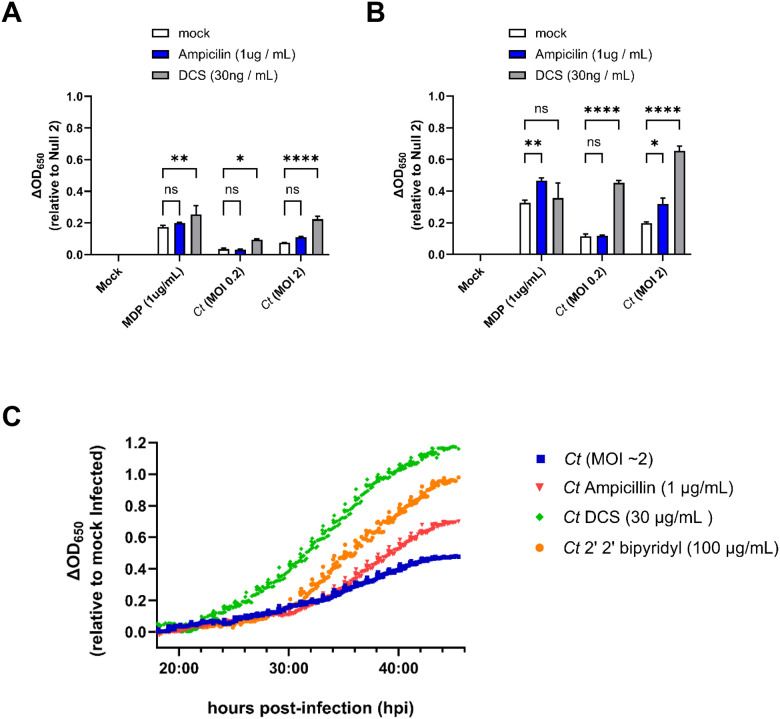
Inducers of persistence enhance Chlamydia-specific NOD2 signaling. SEAP activity was measured from the supernatants of hNOD2 and Null2 HEK 293 reporter cells infected with *C. trachomatis* serovar L2 (strain Bu/434) in the presence / absence of antibiotics that target the assembly (ampicillin) and biosynthesis (D-cycloserine; DCS) of peptidoglycan at 24 **(A)** and 44 **(B)** hpi. Columns represent the mean value calculated for data acquired from three separate experiments (biological replicates) and error bars represent standard error of the mean. Groups were compared via two-way ANOVA with multiple comparisons. ****; p ≤ 0.0001, **; p≤ 0.01, *; p ≤ 0.05, ns; not significant. **(C)** SEAP activity was measured in the supernatants of infected NOD2-expressing HEK 293 reporter cells grown in HEK-Blue^™^ Detection media in the presence / absence of peptidoglycan-targeting antibiotics (ampicillin, DCS), and the iron-chelator 2’2 bipyridyl. OD_650_ values for each well were assessed every ten minutes beginning at 18 hpi, and values plotted are relative to mock infected control wells on the same plate. Data is representative of three technical replicates per group.

Under stress-inducing conditions, chlamydial species are capable of entering a state of persistence in which their replicative forms stop dividing, and the developmental cycle will effective pause until the stress-inducing condition is lifted, after which the microbe will resume dividing and transition into its infectious form^65^. There is considerable debate as to whether this aberrant / persistent state is physiologically relevant in the context of active infections^65–71^, but we have proposed that one of the defining characteristics of this state should be the degree to which it impacts host-microbial interactions at the cellular level^56^. Numerous studies have demonstrated that inducing chlamydial persistence can directly impact the recognition of the organism by various TLRs and NLRs^9,56,72,73^. Given that chlamydia-induced NOD1-signaling has been demonstrated to be impacted in persistently infected cells^56^, we reasoned that NOD2 would be similarly impacted. Upon the addition of D-cycloserine and ampicillin, which respectively inhibit peptidoglycan precursor synthesis^74,75^ and assembly^76^, we found that NOD2 signaling in *C. trachomatis*-infected cells was significantly enhanced (Fig. 4). D-cycloserine was particularly impactful, as NOD2 signaling was observed in DCS-treated, *C. trachomatis*-infected cells as early as 24 hpi (Fig. 4A) and was enhanced as well at later time points (Fig. 4B). When NOD2 signaling was tracked over the span of the chlamydial developmental cycle, signaling from DCS-treated cells began diverging from control and other treatment groups ~23–24 hpi, while ampicillin-treated cells diverged at ~38 hpi (Fig. 4C). We also observed enhanced NOD2 signaling in cells that were treated with the iron chelator 2,2′-bipyridyl, an interesting observation, as this condition has previously been demonstrated to significantly reduce chlamydia-specific NOD1 signaling at early infection time points^56^. Overall, these results demonstrate that induction of aberrance / persistence with the use of PG-targeting antibiotics and iron chelators can drastically impact the degree and kinetics of chlamydia-induced NOD2 signaling.

### Pre-treatment of NOD-expressing HepG2 cells marginally impacts the development of *C. trachomatis*.

**Figure 5.**
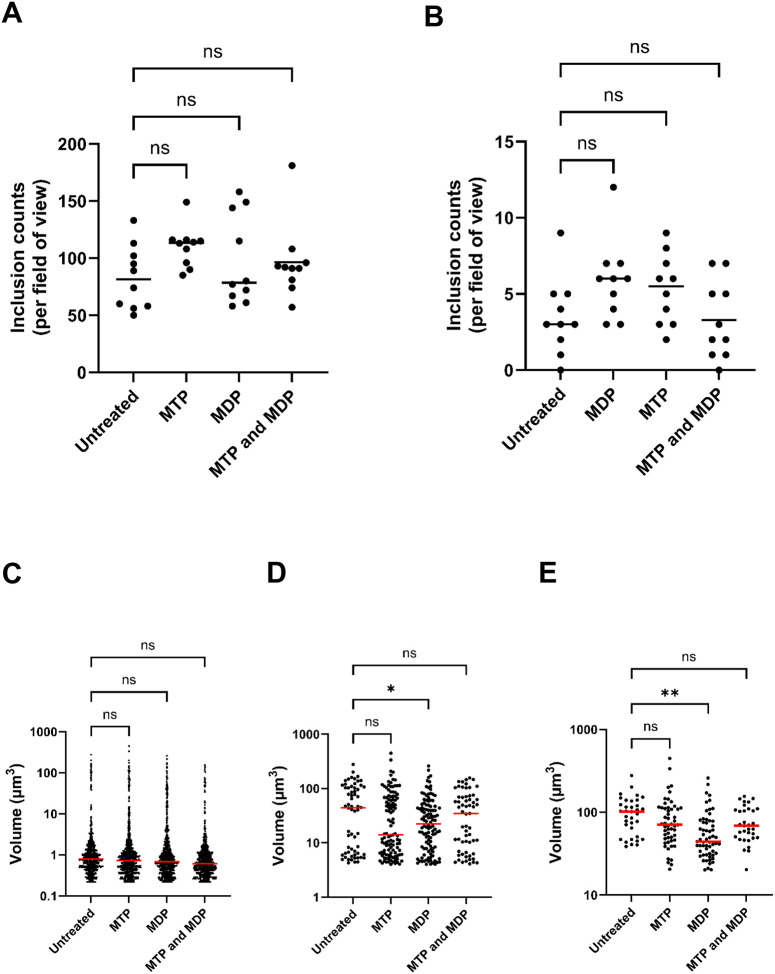
Pre-treatment of NOD1 and NOD2-expressing HepG2 cells marginally impacts the development of *C. trachomatis*. HepG2 cells were pretreated with muramyl tripeptide (MTP), muramyl dipeptide (MDP), or both, and then infected with *C. trachomatis* L2 strain Bu/434. At 24 hpi, cells were fixed, inclusions were labeled, counted, and measured. Data presented represent all MOMP-labeled objects counted **(A)** and those > 20 μm^3^; 3 μm across **(B)** per field of view. Each data point represents a separate field of view counted. Lines represent the mean of the 10 imaging fields counted per group. Significance was assessed via 1-way ANOVA with multiple comparisons. ns; not significant. Approximate inclusion volume measurements were calculated from zStacks of MOMP-labeled objects obtained from a Zeiss 700 confocal microscope. Data is presented for all MOMP-labeled objects observed **(C)**, objects ≥ 4 μm^3^; 2 μm across **(D)** and objects ≥ 20 μm^3^; 3 μm across **(E)**. Each dot represents a single, MOMP-labeled object, and red lines represent the mean of all MOMP-labeled objects within each group. Significance was assessed via 1-way ANOVA with multiple comparisons. **; p ≤ 0.01, *; p ≤ 0.05, ns; not significant.

Given the difference in signaling kinetics observed between NOD1 and NOD2-expressing HEK293 cells infected with *C. trachomatis* and *C. muridarum*, we wanted to examine whether the resulting signaling cascades adversely impact Chlamydial growth / development under native expression conditions. We pre-treated HepG2 cells, which have been previously shown to express both NOD1^77^ and NOD2^78^, with ligands that stimulate NOD1 (L-Ala-γ-D-Glu-mDAP; triDAP) or NOD2 (muramyl dipeptide; MDP) and subsequently infected them with *C. trachomatis* at an MOI of ~4. At 24 hpi, we compared inclusion number and size to untreated controls. Inclusion numbers were roughly equivalent between all control and test groups examined (Fig. 5A, B), indicating that NOD-stimulation did not appear to adversely impact inclusion formation. No significant differences were observed when inclusion size was compared between all groups (Fig. 5C), however, when measurements were limited to inclusions ≥ 4 μm^3^ (~2 μm in diameter) and ≥20 μm^3^ (~3 μm in diameter), cells pretreated with MDP exhibited slightly smaller inclusion sizes when compared to untreated, MTP, and MDP + MTP-treated conditions (Fig. 5D, E).

### Both hNOD2 and mNOD2 recognize C. trachomatis and C. muridarum-specific muropeptides.

**Figure 6.**
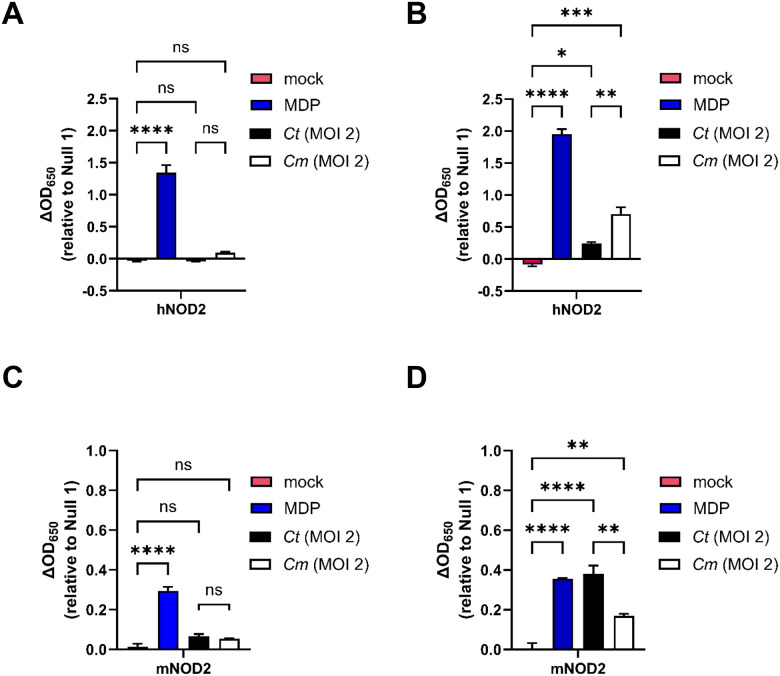
Both hNOD2 and mNOD2 recognize *C. trachomatis* and *C. muridarum*-specific muropeptides. SEAP activity was measured from the supernatants of hNOD2, mNOD2, and Null2 HEK 293 reporter cells infected with *C. trachomatis* serovar L2 (strain Bu/434) at 24 **(A, C)** and 44 **(B, D)** hpi. Columns represent the mean value calculated for data acquired from three separate experiments (biological replicates) and error bars represent standard error of the mean. Groups were compared via one-way ANOVA with multiple comparisons. ****; p ≤ 0.0001, ***; p ≤0.001, **; p ≤ 0.01, *; p ≤ 0.05, ns, not significant.

To conclude this study, we set out to determine whether NOD2 signaling kinetics in Chlamydia-infected cells was unique to i) the human pathogen and/or ii) the human NOD2 receptor. Subsequent experiments were carried out in tandem comparing the NOD2-stimulatory potential of *C. trachomatis* with the murine pathogen *Chlamydia muridarum*. We found that, like *C. trachomatis*, *C. muridarum-*induced hNOD2 expression also appeared to exhibit delayed signaling (Fig. 6A, B). Interestingly, *C. muridarum* also appeared to induce hNOD2 to a greater extent than *C. trachomatis* (Fig. 6B). We hypothesized that this heightened signaling from *C. muridarum* might be due to one of two factors: differences in the rates of replication between the two pathogens^79^ and / or differences in how each pathogen’s PG was recognized by hNOD2. To test these two non-exclusive possibilities, we replicated our signaling study in HEK 293 cells expressing the murine version of the NOD2 receptor (mNOD2). Both pathogens exhibited similar kinetics in mNOD2 signaling, with SEAP activity only observable at the 44 hpi time point (Fig. 6C,D). Surprisingly, *C. trachomatis* induced higher signaling than *C. muridarum* in mNOD2-expressing cells (Fig. 6D), potentially indicating that species-specific recognition, rather than differences in bacterial replication rates, is the driving force behind observed differences in NOD2 signaling between these two species.

## DISCUSSION

We have presented data indicating that NOD1 and NOD2 signaling in Chlamydia-infected cells is temporally distinct, and that the kinetics of signaling appear to be impacted by the mechanism(s) the organism utilizes to degrade its PG-derived muropeptides. While these differences appear to be significant, their potential impact on chlamydial development *in vitro* and *in vivo* is less clear. One potential impact of altering NLR signaling might be modulating signaling cascades in such a manner as to be beneficial to the pathogen. While NLRs are associated with upregulation of various cytokine responses^80^, many of which are prevalent during chlamydial infections^81^, one notable difference between NOD1/2 signaling involves a process of direct relevance to most intracellular pathogens: autophagy. NOD2^−/−^ mice exhibit hypersensitivity to viruses as a result of defects in autophagy, specifically mitophagy, resulting in NLRP3 inflammasome activation and the generation of enhanced levels of IL-18^82^. Conversely, chlamydia-infected cells demonstrate the promotion of autophagy^83–85^ and the inhibition of apoptosis^86–89^. While autophagy has been demonstrated to occur in Chlamydia-infected cells in culture, significant levels are generally not observable until later in chlamydial development^83^ and defects in autophagy have been demonstrated to enhance chlamydial growth^84,85^.

NOD2 is generally thought to enhance cellular autophagy through ATG16L1 in a PI3K, ATG5, and ATG7-dependent manner^90^. ATG16L1 has been shown to restrict the expansion of the chlamydial inclusion, but is re-directed by the secreted chlamydial protein CT622/TaiP to enable vesicular traffic to the inclusion^91^. Given that we see a slight restriction in inclusion expansion in our MDP pre-treated HepG2 cells (Fig. 5D,E), it is tempting to speculate that NOD2 signaling impacts chlamydial development by recruiting the majority of cellular ATG16L1 towards autophagy, thus leaving less free to be utilized by TaiP to recruit vesicular traffic to the growing chlamydial inclusion. The regulation of NOD2-stimulatory ligands by chlamydial species may also exhibit tissue-specific effects, as MDP has recently been shown to increase oxidative respiration and ATP production while decreasing oxidative stress in human intestinal epithelial cells^92^. Experiments investigating these interesting possibilities are currently ongoing.

The observation that *C. muridarum* and *C. trachomatis* appear to differ in the intensity of their signaling in a host-specific manner is another notable finding. While both hNOD2 and mNOD2 recognize MDP and both have similar downstream signaling pathways, some differences have been observed such as the degree to which mutant alleles are capable of suppressing IL-10 expression^93^. Species-specific differences have been noted for other TLRs and NLRs with regard to their regulation, ligand specificity and function^94^, and differences in ligand specificity and sensitivity have been previously demonstrated for both hNOD1 and mNOD1^95^.

As persistence in *Chlamydia* species often leads to a disruption in membrane integrity^96^, we were not surprised by the finding that NOD2 levels increased significantly as a result of persistence induction. The finding that DCS substantially impacts not only the degree but also the timing of Chlamydia-specific NOD2 signaling was particularly interesting. This observation matches those previously made investigating Chlamydia-derived, NOD2-stimulatory ligands present in HeLa cell lysates^57^, indicating that the enhancement is not simply a result of the sonication steps involved in preparing the material for evaluation. DCS is a D-alanine analog, originally isolated from *Streptomyces*^97,98^, and historically its mechanism of action has been understood to be the result of it competitively binding to D-alanine racemase; Alr^75^ and D-alanine-D-alanine ligase; Ddl^99^. Chlamydia species lack canonical Alr and DadX homologs, but encode GlyA enzymes capable of D-Ala racemase activity, which is also inhibited by DCS^100^. The inactivation of these two enzymes should inhibit PG biosynthesis. As chlamydial species do not encode MpaA homologs that would normally function to hydrolyze γ-D-glutamyl-diaminopimelic acid^101^, the result of treatment should be the accumulation of UDP-MurNAc-L-Ala-D-Glu-*meso*-DAP (UDP-MTP) in the chlamydial cytoplasm. While muramyl tripeptide (MTP) is not well recognized by NOD2^95^, the presence of a Uridine Diphosphate (UDP) carrier group in the PG precursor molecule (UDP-MTP) substantially enhances its recognition by NOD2 receptors^102^. This would support the hypothesis that NOD2 signaling from DCS-treated Chlamydia-infected cells is likely due to the release of PG-precursors (namely UDP-MTP) by aberrant RBs. Given that UDP-MTP is normally localized to the bacterial cytoplasm, this would appear to indicate that chlamydial RBs under these conditions are undergoing appreciable levels of cellular lysis. Alternatively, it has been recently suggested that DCS is capable of being utilized as a substrate by both L,D- and D,D- transpeptidases and is capable of being incorporated into the stem peptide of PG at the 4^th^ and 5^th^ position, respectively^103^. It is presently unclear whether these DCS-containing peptide stems are capable of being crosslinked, or whether their presence significantly impacts immunorecognition by NLRs.

The chlamydial amidase AmiA_CT_ appears to meaningfully impact Chlamydia-induced NOD2 signaling (Fig 3). During PG turnover, AmiA_CT_ cleaves stem peptides from MurNAc^61^, and only once this has occurred can lytic transglycosylases (ie. SpoIID_CT_) bind to and subsequently degrade the newly denuded PG glycan stands. However, we have also shown that disruptions in membrane integrity, alterations in PG biosynthesis, and lytic events can all influence chlamydial-derived NOD2 signaling. The enhanced background signaling and slightly enlarged RBs present in our uninduced, AmiA_CT_ knockdown strain would appear to indicate low levels of dCAS9 expression. When baseline NOD2 levels are equivalent between all three strains when using a lower MOI, we see a significant decrease in NOD2 signaling upon induction specific to our knockdown strain (Fig. 3B). Assuming that SpoIID_CT_ is unable to process glycan strands prior to amidase activity, it is somewhat puzzling that the knockdown of amidase activity would impact NOD2 activity. As we observe elevated NOD2 signaling at higher MOIS and lower activity under induction conditions at lower MOIs, we speculate that this data may represent different origins of NOD2-stimulatory ligands and potentially support the hypothesis that PG fragments are not the principle NOD2-stimulatory ligands being released by *C. trachomatis*. Given our DCS-treatment data (Fig. 4), we conclude that PG-precursors that still retain their UDP carrier motif likely make up a substantial portion of the NOD2-stimulatory ligands present in Chlamydia-infected cells, though subsequent experimentation will be needed to further validate this.

**Figure 7.**
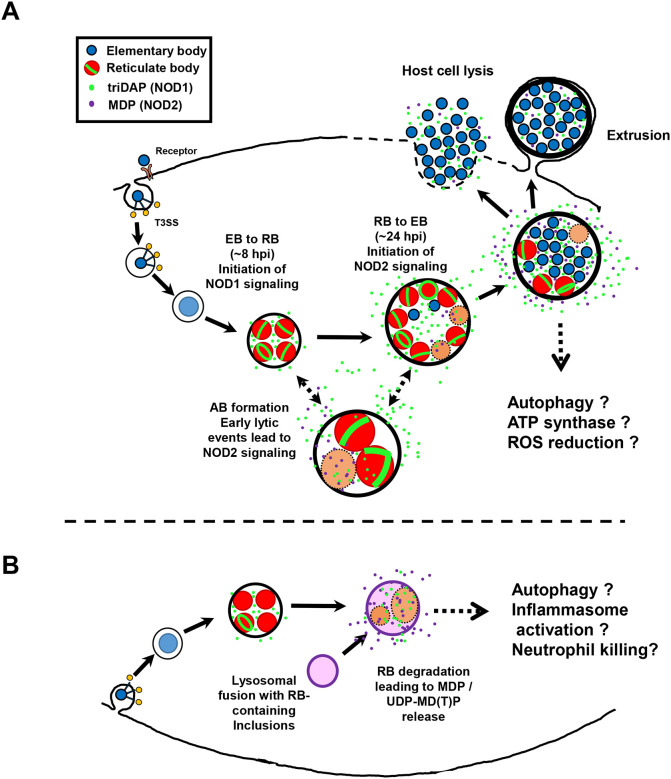
NOD2 signaling as a function of chlamydial RB lysis during the developmental cycle and ‘persistence’. **(A)** During normal developmental conditions, the chlamydial inclusion avoids fusing with lysosomal compartments in the host cell. After RB conversion, the degradation of PG by AmiA_CT_ during chlamydial replication results in the release of NOD1-stimulatory peptides. Lytic events triggered by the RB-to-EB conversion or ‘persistence’ inducers result in the release of partially degraded PG as well as PG precursors, which activate NOD2 signaling. **(B)** In cases were the chlamydial inclusion fuses with lysosomes (such as in phagocytic cells), RBs are degraded and NOD2-stimulatory ligands released at a higher abundance, potentially leading to alterations in inflammasome activation.

While this study effectively demonstrates potential differences in the temporal kinetics of Chlamydia-induced NOD1 and NOD2 signaling, it is important to point out that these phenotypes will almost certainly differ in hematopoietic vs. non-hematopoietic cell types. While *C. trachomatis* may effectively delay NOD2 signaling until late in development by cleaving stem peptides during the natural PG degradative processes associated with its division cycle, this may not significantly impact the production of NOD2-stimulatory PG fragments generated by cellular responses to infection, such as the potential degradation of RBs within lysosomal compartments within hematopoietic cells (Fig. 7). However, as only chlamydial RBs synthesize and maintain PG^13,15^, its availability for NOD1/2 interactions in immune cells would largely depend on the kinetics of the pathogen trafficking to lysosomes. Given that the EB-to-RB transition occurs roughly 8 to 12 hpi, this is presumably plenty of time for EB-containing vesicles to traffic to lysosomes^104,105^. It is tempting to speculate that the origins of EB-to-RB transition kinetics may have arisen, in part, as a means of dampening potential immune responses generated in hematopoietic cells that are incompatible with chlamydial replication and development. Alternatively, as the major NOD2-stimulatory ligand (MDP) is a known activator of NALP3/NLRP3^106^, its release during RB degradation could contribute to neutrophil killing via NLRP3 inflammasome^107^. Work investigating how the timing of inclusion-lysosomal fusion impacts these downstream processes in a cell-type specific manner is currently under active investigation.

## CONCLUSIONS

In this study, we demonstrate that chlamydia infection can be detected by both NOD1 and NOD2 receptors *in vitro*, but that the kinetics of that signaling differs significantly with NOD2 signaling lagging behind that of NOD1. Additionally, we effectively demonstrate that the timing and intensity of NOD2 signaling can be impacted by a variety of factors: chlamydial / host species, incidence of chlamydial lysis, the relative activity of the chlamydial amidase enzyme, and the induction of aberrance / ‘persistence’ phenotypes. Our results strongly suggest that chlamydial PG precursors, rather than degraded PG fragments, are the primary stimulators of NOD2. Given NOD2’s unique association with cellular autophagy induction, we reason that the delay in NOD2-induction by chlamydial species, either directly or indirectly, benefits the development of these obligate, intracellular pathogens.

## MATERIALS AND METHODS

### Reagents:

Anti-MOMP (LS-C123239) was purchased from LSBio. LpxC inhibitor LPC-011 was graciously provided by Dr. Pei Zhou (Duke University).

### Bacterial Strains and Cell Lines:

*C. trachomatis* serovar L2 strain 434/Bu, *C. muridarum* strain Nigg, and *E. coli* strain MG1655 were provided by Anthony Maurelli (University of Florida). *C. trachomatis* and *C. muridarum* stocks were generated utilizing HeLa-USU cells (also provided by Anthony Maurelli) unless otherwise noted. *C. trachomatis* strains -pL2 transformed with pLCria (NT), pLCria (*aimA*), and pLCria-amiA_6xHIS (amiA) vectors were provided by Scot Ouellette (University of Nebraska Medical Center) and are characterized in Dannenberg *et al*. (manuscript in prep). Whole cell lysate (‘crude’) freezer stocks of *C. trachomatis* and *C. muridarum* EBs were generated from HeLa cells 40 hours post infection and stored at −80° C in sucrose phosphate glutamic acid buffer (7.5% w/v sucrose, 17 mM Na_2_HPO_4_, 3 mM NaH_2_PO_4_, 5 mM L-glutamic acid, pH 7.4) until use. Stocks were titered via inclusion forming unit (IFU) assay (described below). HEK-Blue-hNOD1, -hNOD2, -mNOD2, and -Null1 cells were purchased from InvivoGen and propagated according to the manufacturer’s instructions. Cell lines were passaged in high-glucose Dulbecco’s modified Eagle medium (DMEM; Gibco) and 10% fetal bovine serum (FBS; HyClone). HepG2 cells were purchased from ATCC and propagated according to their instructions. All cell lines were checked for mycoplasma contamination 2 passages after the initial liquid nitrogen thaw, and every subsequent 10 passages.

### HEK-Blue hNOD2, mNOD2, Null1 and Null2 NF-κB reporter assay:

HEK-Blue cells expressing human or murine NOD2 and carrying the NF-κB SEAP (secreted embryonic alkaline phosphatase) reporter gene (InvivoGen) were used according to the manufacturer’s instructions and adapted to assess NLR-specific NF-κB activity induced via live *C. trachomatis* and *C. muridarum*. Briefly, 3 × 10^5^ cells/ml were plated in 96-well plates (total reaction volume of 200 μl per well [~6.0 × 10^4^ cells per well]) and allowed to settle/adhere overnight at 37°C. Media was then removed and replaced with 200 μl of medium containing either *C. trachomatis* or *C. muridarum* (at the MOIs indicated) or known NOD-stimulatory ligands (triDAP and MDP). Plates were then centrifuged for 1 h at 2,000 × *g* and subsequently incubated in a CO_2_ incubator at 37°C. Cell supernatants were collected at indicated time points for subsequent analysis of SEAP activity. A colorimetric reporter assay was then utilized to quantify the abundance of SEAP in cell supernatants. Twenty microliters of supernatant collected from infected cells was added to 180 μl of the SEAP detection solution (InvivoGen), followed by incubation at 37°C for ~6 h. SEAP enzymatic activity was then quantified using a plate reader set to 650 nm. Infected cells were compared to uninfected cells (negative control) and cells treated with triDAP / MDP (positive controls). To ensure that changes in alkaline phosphatase activity were NLR-dependent under each of the experimental conditions tested, all experiments were carried out in parallel in either HEK-Blue-Null1 or HEK-Blue-Null2 cell lines, which contains empty expression vectors but lack NOD1/2. Each HEK-Blue SEAP reporter assays was always carried out in three separate experiments, statistical analysis was conducted by either 1- or 2-way ANOVA, and significance values were analyzed by utilizing Sidak’s multiple-comparison test. For real-time tracking of NOD2 signaling, hNOD2 reporter cells were plated, and infected as described above, but instead of DMEM / 10% FBS, cells were incubated in HEK-Blue Detection cell culture medium. Plates were placed in a Stratus microplate reader (Cerillo), which itself was placed in a CO_2_ incubator at 37°C. OD650 readouts were obtained beginning at 18hpi every 10 minutes for the subsequent 28 hours.

### *C. trachomatis* Infections of HepG2 cells:

For infecting HepG2 cells, ~2.5 × 10^5^ cells / mL were spun down and resuspended in infection medium containing ~1×10^6^ IFU of *C. trachomatis* (MOI ~4). 2.5 × 10^5^ cells were then added to each well of a 24 well plate and placed in the incubator for 24 or 44 hours. For pre-stimulation experiments, ~2.5 × 10^5^ cells / mL were spun down and resuspended in infection medium + / − 20 μg/mL triDAP, 20 μg/mL muramyl dipeptide (MDP), or 10 ug/mL of each and incubated overnight at 37° C. The next morning, cells were spun down and resuspended in infection medium containing ~1×10^6^ IFU of *C. trachomatis* (MOI ~4), 2.5 × 10^5^ cells were then added to each well of a 24 well plate and placed in the incubator (at 37° C) for an additional 24 hours.

### Fluorescence Microscopy / MOMP-labeling:

Briefly, HeLa cells were infected with *C. trachomatis* L2 434/Bu as described above. At indicated time points infection medium was removed, and cells were washed three times with 1× PBS. Cells were fixed and permeabilized with methanol for 5 min and gently washed three times with 1× PBS. Cells were then further permeabilized and blocked as described above. Cells were then incubated with anti-MOMP (1:500) for 1 hour at room temperature, followed by several washes in 3% BSA, and subsequent incubation with anti-goat conjugated antibodies (1:1000 Alexa594). For visualizing cell nuclei, cells were incubated with Hoechst stain for ~10 min and then washed with 3% BSA and 1× PBS. Coverslips were mounted on slides with ProLong gold antifade mounting medium and stored in the dark at 4°C prior to imaging via structured-illumination (Elyra PS.1) microscopy. For inclusion size measurements, imaging was conducted on a Zeiss 980 confocal microscope. zStacks were acquired at random for ~10 fields of view per condition, and inclusion volume dimensions were calculated utilizing the 3D object counter addon (ImagJ / Fiji). In order to estimate inclusion diameters, the equations d=2r and r=(3(V/4π))^1/3^ were used with the assumption that inclusions most closely resemble spheres.

### Data Availability:

All data generated in this manuscript will be made available without restriction upon request. This includes all super resolution imaging data, all reporter cell line SEAP data, and chlamydial IFU calculations.

## References

[1] Newman L. Global Estimates of the Prevalence and Incidence of Four Curable Sexually Transmitted Infections in 2012 Based on Systematic Review and Global Reporting. PLoS One 10, e0143304 (2015). 10.1371/journal.pone.0143304 26646541 PMC4672879

[2] Organization, W. H. WHO Alliance for the Global Elimination of Trachoma: progress report on elimination of trachoma, 2023. 363–380 (2024).

[3] Price M. J. The natural history of Chlamydia trachomatis infection in women: a multi-parameter evidence synthesis. Health Technol Assess 20, 1–250 (2016). 10.3310/hta20220 PMC4819202 27007215

[4] Farley T. A., Cohen D. A. & Elkins W. Asymptomatic sexually transmitted diseases: the case for screening. Prev Med 36, 502–509 (2003). 10.1016/s0091-7435(02)00058-0 12649059

[5] Elwell C., Mirrashidi K. & Engel J. Chlamydia cell biology and pathogenesis. Nature reviews. Microbiology 14, 385–400 (2016). 10.1038/nrmicro.2016.30 27108705 PMC4886739

[6] Omsland A., Sager J., Nair V., Sturdevant D. E. & Hackstadt T. Developmental stage-specific metabolic and transcriptional activity of Chlamydia trachomatis in an axenic medium. Proceedings of the National Academy of Sciences of the United States of America 109, 19781–19785 (2012). 10.1073/pnas.1212831109 23129646 PMC3511728

[7] Ingalls R. R. The inflammatory cytokine response to Chlamydia trachomatis infection is endotoxin mediated. Infect Immun 63, 3125–3130 (1995). 10.1128/iai.63.8.3125-3130.1995 7542638 PMC173426

[8] Yang C. Chlamydia trachomatis Lipopolysaccharide Evades the Canonical and Noncanonical Inflammatory Pathways To Subvert Innate Immunity. mBio 10 (2019). 10.1128/mBio.00595-19 PMC6479002 31015326

[9] Diallo A., Overman G., Sah P. & Liechti G. W. Recognition of Chlamydia trachomatis by Toll-like receptor 9 is altered during persistence. Infect Immun 92, e0006324 (2024). 10.1128/iai.00063-24 38899879 PMC11238561

[10] Ouburg S. TLR9 KO mice, haplotypes and CPG indices in Chlamydia trachomatis infection. Drugs Today (Barc) 45 Suppl B, 83–93 (2009). 20011699

[11] Singer M., de Waaij D. J., Morre S. A. & Ouburg S. CpG DNA analysis of bacterial STDs. BMC Infect Dis 15, 273 (2015). 10.1186/s12879-015-1016-7 26179610 PMC4504089

[12] Abdelrahman Y., Ouellette S. P., Belland R. J. & Cox J. V. Polarized Cell Division of Chlamydia trachomatis. PLoS Pathog 12, e1005822 (2016). 10.1371/journal.ppat.1005822 27505160 PMC4978491

[13] Liechti G. W. A new metabolic cell-wall labelling method reveals peptidoglycan in Chlamydia trachomatis. Nature 506, 507–510 (2014). 10.1038/nature12892 24336210 PMC3997218

[14] Jacquier N., Viollier P. H. & Greub G. The role of peptidoglycan in chlamydial cell division: towards resolving the chlamydial anomaly. FEMS Microbiol Rev 39, 262–275 (2015). 10.1093/femsre/fuv001 25670734

[15] Liechti G. Pathogenic Chlamydia Lack a Classical Sacculus but Synthesize a Narrow, Mid-cell Peptidoglycan Ring, Regulated by MreB, for Cell Division. PLoS Pathog 12, e1005590 (2016). 10.1371/journal.ppat.1005590 27144308 PMC4856321

[16] Pilhofer M. Discovery of chlamydial peptidoglycan reveals bacteria with murein sacculi but without FtsZ. Nat Commun 4, 2856 (2013). 10.1038/ncomms3856 24292151 PMC3847603

[17] Navarro P. P. Cell wall synthesis and remodelling dynamics determine division site architecture and cell shape in Escherichia coli. Nat Microbiol 7, 1621–1634 (2022). 10.1038/s41564-022-01210-z 36097171 PMC9519445

[18] Turner R. D., Hurd A. F., Cadby A., Hobbs J. K. & Foster S. J. Cell wall elongation mode in Gram-negative bacteria is determined by peptidoglycan architecture. Nat Commun 4, 1496 (2013). 10.1038/ncomms2503 23422664 PMC3586723

[19] Wang S., Furchtgott L., Huang K. C. & Shaevitz J. W. Helical insertion of peptidoglycan produces chiral ordering of the bacterial cell wall. Proceedings of the National Academy of Sciences of the United States of America 109, E595–604 (2012). 10.1073/pnas.1117132109 22343529 PMC3309786

[20] Brown W. J. & Rockey D. D. Identification of an antigen localized to an apparent septum within dividing chlamydiae. Infect Immun 68, 708–715 (2000). 10.1128/IAI.68.2.708-715.2000 10639437 PMC97196

[21] Ouellette S. P., Lee J. & Cox J. V. Division without Binary Fission: Cell Division in the FtsZ-Less Chlamydia. J Bacteriol 202 (2020). 10.1128/JB.00252-20 PMC7417837 32540934

[22] Barrows J. M. & Goley E. D. FtsZ dynamics in bacterial division: What, how, and why? Curr Opin Cell Biol 68, 163–172 (2021). 10.1016/j.ceb.2020.10.013 33220539 PMC7925355

[23] Gaballah A., Kloeckner A., Otten C., Sahl H. G. & Henrichfreise B. Functional analysis of the cytoskeleton protein MreB from Chlamydophila pneumoniae. PLoS One 6, e25129 (2011). 10.1371/journal.pone.0025129 22022378 PMC3187750

[24] Ouellette S. P., Karimova G., Subtil A. & Ladant D. Chlamydia co-opts the rod shape-determining proteins MreB and Pbp2 for cell division. Mol Microbiol 85, 164–178 (2012). 10.1111/j.1365-2958.2012.08100.x 22624979

[25] Ouellette S. P. Analysis of MreB interactors in Chlamydia reveals a RodZ homolog but fails to detect an interaction with MraY. Front Microbiol 5, 279 (2014). 10.3389/fmicb.2014.00279 24936201 PMC4047632

[26] Jacquier N., Frandi A., Pillonel T., Viollier P. H. & Greub G. Cell wall precursors are required to organize the chlamydial division septum. Nat Commun 5, 3578 (2014). 10.1038/ncomms4578 24709914 PMC3988822

[27] Kemege K. E. Chlamydia trachomatis protein CT009 is a structural and functional homolog to the key morphogenesis component RodZ and interacts with division septal plane localized MreB. Mol Microbiol 95, 365–382 (2015). 10.1111/mmi.12855 25382739 PMC4485377

[28] Ouellette S. P., Rueden K. J., AbdelRahman Y. M., Cox J. V. & Belland R. J. Identification and Partial Characterization of Potential FtsL and FtsQ Homologs of Chlamydia. Front Microbiol 6, 1264 (2015). 10.3389/fmicb.2015.01264 26617598 PMC4643143

[29] Harpring M., Lee J., Zhong G., Ouellette S. P. & Cox J. V. FtsK is Critical for the Assembly of the Unique Divisome Complex of the FtsZ-less Chlamydia trachomatis. bioRxiv (2025). 10.1101/2024.10.24.620021 PMC11975371 40193186

[30] Meeske A. J. SEDS proteins are a widespread family of bacterial cell wall polymerases. Nature 537, 634–638 (2016). 10.1038/nature19331 27525505 PMC5161649

[31] Taguchi A. FtsW is a peptidoglycan polymerase that is functional only in complex with its cognate penicillin-binding protein. Nat Microbiol 4, 587–594 (2019). 10.1038/s41564-018-0345-x 30692671 PMC6430707

[32] Cox J. V., Abdelrahman Y. M. & Ouellette S. P. Penicillin-binding proteins regulate multiple steps in the polarized cell division process of Chlamydia. Sci Rep 10, 12588 (2020). 10.1038/s41598-020-69397-x 32724139 PMC7387471

[33] Liechti G. W. Localized Peptidoglycan Biosynthesis in Chlamydia trachomatis Conforms to the Polarized Division and Cell Size Reduction Developmental Models. Front Microbiol 12, 733850 (2021). 10.3389/fmicb.2021.733850 34956109 PMC8699169

[34] Wong W. F., Chambers J. P., Gupta R. & Arulanandam B. P. Chlamydia and Its Many Ways of Escaping the Host Immune System. J Pathog 2019, 8604958 (2019). 10.1155/2019/8604958 31467721 PMC6699355

[35] Chen H., Wen Y. & Li Z. Clear Victory for Chlamydia: The Subversion of Host Innate Immunity. Front Microbiol 10, 1412 (2019). 10.3389/fmicb.2019.01412 31333596 PMC6619438

[36] Wolf A. J. & Underhill D. M. Peptidoglycan recognition by the innate immune system. Nat Rev Immunol 18, 243–254 (2018). 10.1038/nri.2017.136 29292393

[37] Singh R., Liechti G., Slade J. A. & Maurelli A. T. Chlamydia trachomatis Oligopeptide Transporter Performs Dual Functions of Oligopeptide Transport and Peptidoglycan Recycling. Infect Immun 88 (2020). 10.1128/IAI.00086-20 PMC7171251 32094256

[38] Reuter J. An NlpC/P60 protein catalyzes a key step in peptidoglycan recycling at the intersection of energy recovery, cell division and immune evasion in the intracellular pathogen Chlamydia trachomatis. PLoS Pathog 19, e1011047 (2023). 10.1371/journal.ppat.1011047 36730465 PMC9928106

[39] Franchi L., Warner N., Viani K. & Nunez G. Function of Nod-like receptors in microbial recognition and host defense. Immunol Rev 227, 106–128 (2009). 10.1111/j.1600-065X.2008.00734.x 19120480 PMC2679989

[40] Girardin S. E. Nod1 detects a unique muropeptide from gram-negative bacterial peptidoglycan. Science 300, 1584–1587 (2003). 10.1126/science.1084677 12791997

[41] Girardin S. E. Nod2 is a general sensor of peptidoglycan through muramyl dipeptide (MDP) detection. J Biol Chem 278, 8869–8872 (2003). 10.1074/jbc.C200651200 12527755

[42] Zou Y., Lei W., He Z. & Li Z. The role of NOD1 and NOD2 in host defense against chlamydial infection. FEMS Microbiol Lett 363 (2016). 10.1093/femsle/fnw170 27421958

[43] Brankovic I. NOD1 in contrast to NOD2 functional polymorphism influence Chlamydia trachomatis infection and the risk of tubal factor infertility. Pathog Dis 73, 1–9 (2015). 10.1093/femspd/ftu028 PMC4542905 25854006

[44] Jukema J. B. Can Previous Associations of Single Nucleotide Polymorphisms in the TLR2, NOD1, CXCR5, and IL10 Genes in the Susceptibility to and Severity of Chlamydia trachomatis Infections Be Confirmed? Pathogens 10 (2021). 10.3390/pathogens10010048 PMC7827792 33430411

[45] den Hartog J. E. Do host genetic traits in the bacterial sensing system play a role in the development of Chlamydia trachomatis-associated tubal pathology in subfertile women? BMC Infect Dis 6, 122 (2006). 10.1186/1471-2334-6-122 16859562 PMC1555588

[46] Hart K. M. Functional expression of pattern recognition receptors in tissues of the human female reproductive tract. J Reprod Immunol 80, 33–40 (2009). 10.1016/j.jri.2008.12.004 19406482 PMC2744441

[47] Hafner L. M., Cunningham K. & Beagley K. W. Ovarian steroid hormones: effects on immune responses and Chlamydia trachomatis infections of the female genital tract. Mucosal Immunol 6, 859–875 (2013). 10.1038/mi.2013.46 23860476

[48] Lebedeva O. P. The role of NOD1 and NOD2 receptors in recognizing pathogens in the female reproductive tract. Obstetrics and Gynecology, 25–29 (2019).

[49] Buchholz K. R. & Stephens R. S. The cytosolic pattern recognition receptor NOD1 induces inflammatory interleukin-8 during Chlamydia trachomatis infection. Infect Immun 76, 3150–3155 (2008). 10.1128/IAI.00104-08 18426885 PMC2446689

[50] Opitz B. Nod1-mediated endothelial cell activation by Chlamydophila pneumoniae. Circ Res 96, 319–326 (2005). 10.1161/01.RES.0000155721.83594.2c 15653568

[51] Welter-Stahl L. Stimulation of the cytosolic receptor for peptidoglycan, Nod1, by infection with Chlamydia trachomatis or Chlamydia muridarum. Cell Microbiol 8, 1047–1057 (2006). 10.1111/j.1462-5822.2006.00686.x 16681844

[52] Pham O. H. NOD1/NOD2 and RIP2 Regulate Endoplasmic Reticulum Stress-Induced Inflammation during Chlamydia Infection. mBio 11 (2020). 10.1128/mBio.00979-20 PMC7267884 32487756

[53] Shimada K. The NOD/RIP2 pathway is essential for host defenses against Chlamydophila pneumoniae lung infection. PLoS Pathog 5, e1000379 (2009). 10.1371/journal.ppat.1000379 19360122 PMC2660273

[54] Keestra-Gounder A. M. NOD1 and NOD2 signalling links ER stress with inflammation. Nature 532, 394–397 (2016). 10.1038/nature17631 27007849 PMC4869892

[55] Belland R. J. Genomic transcriptional profiling of the developmental cycle of Chlamydia trachomatis. Proceedings of the National Academy of Sciences of the United States of America 100, 8478–8483 (2003). 10.1073/pnas.1331135100 12815105 PMC166254

[56] Brockett M. R. & Liechti G. W. Persistence alters the interaction between Chlamydia trachomatis and its host cell. Infect Immun (2021). 10.1128/IAI.00685-20 PMC8281235 34001559

[57] Packiam M., Weinrick B., Jacobs W. R. Jr. & Maurelli A. T. Structural characterization of muropeptides from Chlamydia trachomatis peptidoglycan by mass spectrometry resolves “chlamydial anomaly”. Proceedings of the National Academy of Sciences of the United States of America 112, 11660–11665 (2015). 10.1073/pnas.1514026112 26290580 PMC4577195

[58] Lee J. K. Replication-dependent size reduction precedes differentiation in Chlamydia trachomatis. Nat Commun 9, 45 (2018). 10.1038/s41467-017-02432-0 29298975 PMC5752669

[59] Cram E. D., Rockey D. D. & Dolan B. P. Chlamydia spp. development is differentially altered by treatment with the LpxC inhibitor LPC-011. BMC Microbiol 17, 98 (2017). 10.1186/s12866-017-0992-8 28438125 PMC5402638

[60] Nguyen B. D. Lipooligosaccharide is required for the generation of infectious elementary bodies in Chlamydia trachomatis. Proceedings of the National Academy of Sciences of the United States of America 108, 10284–10289 (2011). 10.1073/pnas.1107478108 21628561 PMC3121853

[61] Klockner A. AmiA is a penicillin target enzyme with dual activity in the intracellular pathogen Chlamydia pneumoniae. Nat Commun 5, 4201 (2014). 10.1038/ncomms5201 24953137 PMC4083426

[62] Jacquier N. A SpoIID Homolog Cleaves Glycan Strands at the Chlamydial Division Septum. mBio 10 (2019). 10.1128/mBio.01128-19 PMC6635528 31311880

[63] Adamson C., Liang Y., Feng S., Ng A. W. R. & Qiao Y. A closer look at ligand specificity for cellular activation of NOD2 with synthetic muramyl dipeptide analogues. Chem Commun (Camb) 60, 2212–2215 (2024). 10.1039/d3cc05807g 38305731

[64] Ouellette S. P., Blay E. A., Hatch N. D. & Fisher-Marvin L. A. CRISPR Interference To Inducibly Repress Gene Expression in Chlamydia trachomatis. Infect Immun 89, e0010821 (2021). 10.1128/IAI.00108-21 33875479 PMC8373233

[65] Panzetta M. E., Valdivia R. H. & Saka H. A. Chlamydia Persistence: A Survival Strategy to Evade Antimicrobial Effects in-vitro and in-vivo. Front Microbiol 9, 3101 (2018). 10.3389/fmicb.2018.03101 30619180 PMC6299033

[66] Bavoil P. M. What’s in a word: the use, misuse, and abuse of the word “persistence” in Chlamydia biology. Front Cell Infect Microbiol 4, 27 (2014). 10.3389/fcimb.2014.00027 24624366 PMC3940941

[67] Borel N., Pospischil A., Hudson A. P., Rupp J. & Schoborg R. V. The role of viable but non-infectious developmental forms in chlamydial biology. Front Cell Infect Microbiol 4, 97 (2014). 10.3389/fcimb.2014.00097 25105096 PMC4109588

[68] Wyrick P. B. Chlamydia trachomatis persistence in vitro: an overview. J Infect Dis 201 Suppl 2, S88–95 (2010). 10.1086/652394 20470046 PMC2878585

[69] Workowski K. A., Lampe M. F., Wong K. G., Watts M. B. & Stamm W. E. Long-term eradication of Chlamydia trachomatis genital infection after antimicrobial therapy. Evidence against persistent infection. JAMA 270, 2071–2075 (1993). 8305018

[70] Kozusnik T., Adams S. E. & Greub G. Aberrant Bodies: An Alternative Metabolic Homeostasis Allowing Survivability? Microorganisms 12 (2024). 10.3390/microorganisms12030495 PMC10972484 38543546

[71] Smith E. P. & Valdivia R. H. Chlamydia trachomatis: a model for intracellular bacterial parasitism. J Bacteriol, e0036124 (2025). 10.1128/jb.00361-24 39976429 PMC11925236

[72] O’Connell C. M., Ionova I. A., Quayle A. J., Visintin A. & Ingalls R. R. Localization of TLR2 and MyD88 to Chlamydia trachomatis inclusions. Evidence for signaling by intracellular TLR2 during infection with an obligate intracellular pathogen. J Biol Chem 281, 1652–1659 (2006). 10.1074/jbc.M510182200 16293622

[73] Yu P. STAT3-mediated TLR2/4 pathway upregulation in an IFN-gamma-induced Chlamydia trachomatis persistent infection model. Pathog Dis 74 (2016). 10.1093/femspd/ftw076 27502695

[74] David H. L., Takayama K. & Goldman D. S. Susceptibility of mycobacterial D-alanyl-D-alanine synthetase to D-cycloserine. Am Rev Respir Dis 100, 579–581 (1969). 10.1164/arrd.1969.100.4.579 4981706

[75] Lambert M. P. & Neuhaus F. C. Mechanism of D-cycloserine action: alanine racemase from Escherichia coli W. J Bacteriol 110, 978–987 (1972). 10.1128/jb.110.3.978-987.1972 4555420 PMC247518

[76] Tipper D. J. & Strominger J. L. Mechanism of action of penicillins: a proposal based on their structural similarity to acyl-D-alanyl-D-alanine. Proceedings of the National Academy of Sciences of the United States of America 54, 1133–1141 (1965). 10.1073/pnas.54.4.1133 5219821 PMC219812

[77] Gulzar F. ER stress aggravates NOD1-mediated inflammatory response leading to impaired nutrient metabolism in hepatoma cells. Biochem Biophys Res Commun 735, 150827 (2024). 10.1016/j.bbrc.2024.150827 39423570

[78] Body-Malapel M. NOD2: a potential target for regulating liver injury. Lab Invest 88, 318–327 (2008). 10.1038/labinvest.3700716 18227809

[79] Lyons J. M., Ito J. I. Jr., Pena A. S. & Morre S. A. Differences in growth characteristics and elementary body associated cytotoxicity between Chlamydia trachomatis oculogenital serovars D and H and Chlamydia muridarum. J Clin Pathol 58, 397–401 (2005). 10.1136/jcp.2004.021543 15790704 PMC1770636

[80] Kienes I., Weidl T., Mirza N., Chamaillard M. & Kufer T. A. Role of NLRs in the Regulation of Type I Interferon Signaling, Host Defense and Tolerance to Inflammation. Int J Mol Sci 22 (2021). 10.3390/ijms22031301 PMC7865845 33525590

[81] Murray S. M. & McKay P. F. Chlamydia trachomatis: Cell biology, immunology and vaccination. Vaccine 39, 2965–2975 (2021). 10.1016/j.vaccine.2021.03.043 33771390

[82] Lupfer C. Receptor interacting protein kinase 2-mediated mitophagy regulates inflammasome activation during virus infection. Nat Immunol 14, 480–488 (2013). 10.1038/ni.2563 23525089 PMC3631456

[83] Pachikara N., Zhang H., Pan Z., Jin S. & Fan H. Productive Chlamydia trachomatis lymphogranuloma venereum 434 infection in cells with augmented or inactivated autophagic activities. FEMS Microbiol Lett 292, 240–249 (2009). 10.1111/j.1574-6968.2009.01494.x 19187200 PMC2671565

[84] Al-Younes H. M. Autophagy-independent function of MAP-LC3 during intracellular propagation of Chlamydia trachomatis. Autophagy 7, 814–828 (2011). 10.4161/auto.7.8.15597 21464618

[85] Yasir M., Pachikara N. D., Bao X., Pan Z. & Fan H. Regulation of chlamydial infection by host autophagy and vacuolar ATPase-bearing organelles. Infect Immun 79, 4019–4028 (2011). 10.1128/IAI.05308-11 21807906 PMC3187247

[86] Waguia Kontchou C. Chlamydia trachomatis inhibits apoptosis in infected cells by targeting the pro-apoptotic proteins Bax and Bak. Cell Death Differ 29, 2046–2059 (2022). 10.1038/s41418-022-00995-0 35397654 PMC9525694

[87] Sharma M. & Rudel T. Apoptosis resistance in Chlamydia-infected cells: a fate worse than death? FEMS Immunol Med Microbiol 55, 154–161 (2009). 10.1111/j.1574-695X.2008.00515.x 19281566

[88] Fan T. Inhibition of apoptosis in chlamydia-infected cells: blockade of mitochondrial cytochrome c release and caspase activation. The Journal of experimental medicine 187, 487–496 (1998). 10.1084/jem.187.4.487 9463399 PMC2212145

[89] Dean D. & Powers V. C. Persistent Chlamydia trachomatis infections resist apoptotic stimuli. Infect Immun 69, 2442–2447 (2001). 10.1128/IAI.69.4.2442-2447.2001 11254605 PMC98177

[90] Henderson P. & Stevens C. The role of autophagy in Crohn’s disease. Cells 1, 492–519 (2012). 10.3390/cells1030492 24710487 PMC3901108

[91] Hamaoui D. The Chlamydia effector CT622/TaiP targets a nonautophagy related function of ATG16L1. Proceedings of the National Academy of Sciences of the United States of America 117, 26784–26794 (2020). 10.1073/pnas.2005389117 33055216 PMC7604492

[92] Tian D., Cui M. & Han M. Bacterial muropeptides promote OXPHOS and suppress mitochondrial stress in mammals. Cell Rep 43, 114067 (2024). 10.1016/j.celrep.2024.114067 38583150 PMC11107371

[93] Noguchi E., Homma Y., Kang X., Netea M. G. & Ma X. A Crohn’s disease-associated NOD2 mutation suppresses transcription of human IL10 by inhibiting activity of the nuclear ribonucleoprotein hnRNP-A1. Nat Immunol 10, 471–479 (2009). 10.1038/ni.1722 19349988 PMC2928218

[94] Ariffin J. K. & Sweet M. J. Differences in the repertoire, regulation and function of Toll-like Receptors and inflammasome-forming Nod-like Receptors between human and mouse. Curr Opin Microbiol 16, 303–310 (2013). 10.1016/j.mib.2013.03.002 23540353

[95] Magalhaes J. G. Murine Nod1 but not its human orthologue mediates innate immune detection of tracheal cytotoxin. EMBO Rep 6, 1201–1207 (2005). 10.1038/sj.embor.7400552 16211083 PMC1369207

[96] Frohlich K. M. Membrane vesicle production by Chlamydia trachomatis as an adaptive response. Front Cell Infect Microbiol 4, 73 (2014). 10.3389/fcimb.2014.00073 24959424 PMC4050530

[97] Harned R. L., Hidy P. H. & La Baw E. K. Cycloserine. I. A preliminary report. Antibiot Chemother (Northfield) 5, 204–205 (1955). 24543467

[98] Epstein I. G., Nair K. G. & Boyd L. J. Cycloserine, a new antibiotic, in the treatment of human pulmonary tuberculosis: a preliminary report. Antibiotic Med Clin Ther (New York) 1, 80–93 (1955). 14362439

[99] Prosser G. A. & de Carvalho L. P. Kinetic mechanism and inhibition of Mycobacterium tuberculosis D-alanine:D-alanine ligase by the antibiotic D-cycloserine. FEBS J 280, 1150–1166 (2013). 10.1111/febs.12108 23286234

[100] De Benedetti S. Characterization of serine hydroxymethyltransferase GlyA as a potential source of D-alanine in Chlamydia pneumoniae. Front Cell Infect Microbiol 4, 19 (2014). 10.3389/fcimb.2014.00019 24616885 PMC3935232

[101] Stephens R. S. Genome sequence of an obligate intracellular pathogen of humans: Chlamydia trachomatis. Science 282, 754–759 (1998). 10.1126/science.282.5389.754 9784136

[102] Girardin S. E. Peptidoglycan molecular requirements allowing detection by Nod1 and Nod2. J Biol Chem 278, 41702–41708 (2003). 10.1074/jbc.M307198200 12871942

[103] Kuru E. Mechanisms of Incorporation for D-Amino Acid Probes That Target Peptidoglycan Biosynthesis. ACS Chem Biol 14, 2745–2756 (2019). 10.1021/acschembio.9b00664 31743648 PMC6929685

[104] Sun H. S. Chlamydia trachomatis vacuole maturation in infected macrophages. J Leukoc Biol 92, 815–827 (2012). 10.1189/jlb.0711336 22807527 PMC4050525

[105] Al-Zeer M. A., Al-Younes H. M., Lauster D., Abu Lubad M. & Meyer T. F. Autophagy restricts Chlamydia trachomatis growth in human macrophages via IFNG-inducible guanylate binding proteins. Autophagy 9, 50–62 (2013). 10.4161/auto.22482 23086406 PMC3542218

[106] Faustin B. Reconstituted NALP1 inflammasome reveals two-step mechanism of caspase-1 activation. Mol Cell 25, 713–724 (2007). 10.1016/j.molcel.2007.01.032 17349957

[107] Yang C. Chlamydia evasion of neutrophil host defense results in NLRP3 dependent myeloid-mediated sterile inflammation through the purinergic P2X7 receptor. Nat Commun 12, 5454 (2021). 10.1038/s41467-021-25749-3 34526512 PMC8443728

